# Breed-specific reference sequence optimized mapping accuracy of NGS analyses for pigs

**DOI:** 10.1186/s12864-021-08030-1

**Published:** 2021-10-12

**Authors:** Dan Wang, Liu Yang, Chao Ning, Jian-Feng Liu, Xingbo Zhao

**Affiliations:** 1 National Engineering Laboratory for Animal Breeding, Ministry of Agricultural Key Laboratory of Animal Genetics, Breeding and Reproduction, College of Animal Science and Technology, China Agricultural University, Beijing, China; 2 College of Animal Science and Technology, Shandong Agricultural University, Tai’an, China

**Keywords:** Mitochondrial genome, Mapping, Reference sequence, SNP calling, Pig

## Abstract

**Background:**

Reference sequences play a vital role in next-generation sequencing (NGS), impacting mapping quality during genome analyses. However, reference genomes usually do not represent the full range of genetic diversity of a species as a result of geographical divergence and independent demographic events of different populations. For the mitochondrial genome (mitogenome), which occurs in high copy numbers in cells and is strictly maternally inherited, an optimal reference sequence has the potential to make mitogenome alignment both more accurate and more efficient. In this study, we used three different types of reference sequences for mitogenome mapping, i.e., the commonly used reference sequence (CU-ref), the breed-specific reference sequence (BS-ref) and the sample-specific reference sequence (SS-ref), respectively, and compared the accuracy of mitogenome alignment and SNP calling among them, for the purpose of proposing the optimal reference sequence for mitochondrial DNA (mtDNA) analyses of specific populations

**Results:**

Four pigs, representing three different breeds, were high-throughput sequenced, subsequently mapping reads to the reference sequences mentioned above, resulting in a largest mapping ratio and a deepest coverage without increased running time when aligning reads to a BS-ref. Next, single nucleotide polymorphism (SNP) calling was carried out by 18 detection strategies with the three tools SAMtools, VarScan and GATK with different parameters, using the bam results mapping to BS-ref. The results showed that all eighteen strategies achieved the same high specificity and sensitivity, which suggested a high accuracy of mitogenome alignment by the BS-ref because of a low requirement for SNP calling tools and parameter choices.

**Conclusions:**

This study showed that different reference sequences representing different genetic relationships to sample reads influenced mitogenome alignment, with the breed-specific reference sequences being optimal for mitogenome analyses, which provides a refined processing perspective for NGS data.

**Supplementary Information:**

The online version contains supplementary material available at 10.1186/s12864-021-08030-1.

## Background

Next-generation sequencing (NGS) technology is characterized by providing millions of DNA sequencing reads at a time, with high efficiency and low costs compared to Sanger sequencing [1]. The mitochondrion contains hundreds of mitochondrial genome copies [2], which are, in contrast to the biparentally inherited nuclear genome, strictly maternally inherited. With NGS data, complete mitogenomes have been obtained for many vertebrate species, including pigs [3–6], chickens [7–9] and cattle [10–12]. It has been reported that the reference sequences used affect the accuracy of genome mapping [13–15]. Reference genomes often cannot represent the full range of genetic diversity of a species as a result of geographical divergence and independent demographic events in different populations. To comprehensively characterize genetic variation, different references for different populations may be necessary. This perspective has fueled the research on pan-genomes, currently focusing on nuclear genomes, and not involving mitogenomes [16, 17]. This lack of studies on the influence of reference sequences on mitogenome alignment requires addressing.

In this study, we explored whether the genetic relationships between reference and sample sequence would impact mapping accuracy of pig mitogenomes. Three breeds of pigs, including one Asian wild boar, two unrelated Diannan small-ear pigs and one Tibetan pig, were sampled and high-throughput sequenced. The sequence data were then analysed by genome alignment and SNP calling. We tested the accuracy of mitogenome alignment and SNP calling based on three kinds of reference sequences, which represent three kinds of genetic relationships to samples. The reference sequences included the commonly used reference sequence (CU-ref), breed-specific reference sequences (BS-ref) and sample-specific reference sequences (SS-ref). In detail, (1) the commonly used reference sequence (CU-ref) refers to a frequently-used sequence from the RefSeq project at NCBI database. For pigs, CU-ref is normally the mitogenome sequence from a Landrace pig (NC_000845.1) [18–21]. (2) the breed-specific reference sequence (BS-ref), which refers to a sequence of the same breed as the sample, was downloaded from NCBI database. (3) the sample-specific reference sequence (SS-ref), which refers to the consensus sequence, was obtained from the NGS reads of the sample through de novo assembly. Thereinto, for an optimal de novo assembly for the SS-ref sequence, three levels of NGS read sets, namely, all clean read sets, homologous read sets filtered by BLAST, or filtered by BWA mapping, were used and compared in the de novo assembly software SOAPdenove2 [22] by default parameters with the best k-mer size estimated by KmerGennie [23].

## Results

### Performance of de novo assembly strategies

In order to produce the optimal SS-ref sequences, three de novo assembly strategies were carried out, including the Denovo strategy, the BLAST_denovo strategy, and the BWA_denovo strategy. The BLAST_denovo strategy got a similar result in N50 contig size, consensus length, and genome coverage compared to the Denovo strategy. However, the former strategy yielded no polymorphic site, while the latter created a large number of polymorphisms (193 sites), potentially caused by nuclear mitochondrial sequences (NUMTs). In addition, the third assembly strategy (BWA_denovo) got the smallest N50 contig size and some polymorphic sites. Therefore, the de novo assembly by homologous sequences filtered by BLAST from NGS data was selected for constructing the SS-ref sequence for each sample. NGS data information is listed in Additional file 1: Table S1, and the assembly quality of each de novo strategy is shown in Table 1.

**Table 1:** Statistics of A1 mitogenome assembly by three strategies

Methods	BLAST_denovo	BWA_denovo	Denovo
**best k**	31	99	99
**N50**	1437	112	1867
**consensus length**	16,596	16,598	16,607
**coverage**	99.90%	99.91%	99.96%
**polymorphic sites**	0	2	193

### Alignment quality by different reference sequences

**Fig. 1:**
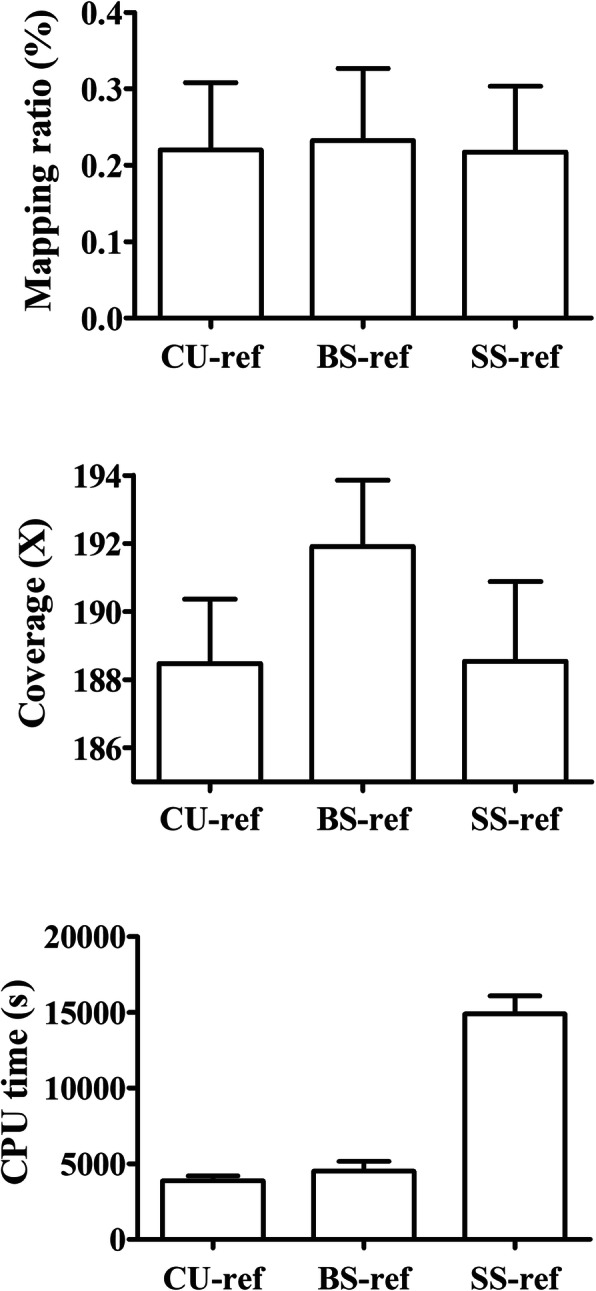
Comparison of mapping ratio (%), coverage (X) and CPU time cost (s) among the three alignment strategies. The three alignment strategies were based on three references with different genetic relationships to samples, i.e., the commonly used reference sequence (CU-ref), the breed-specific reference sequence (BS-ref) and the sample-specific reference sequence (SS-ref)

**Fig. 2:**
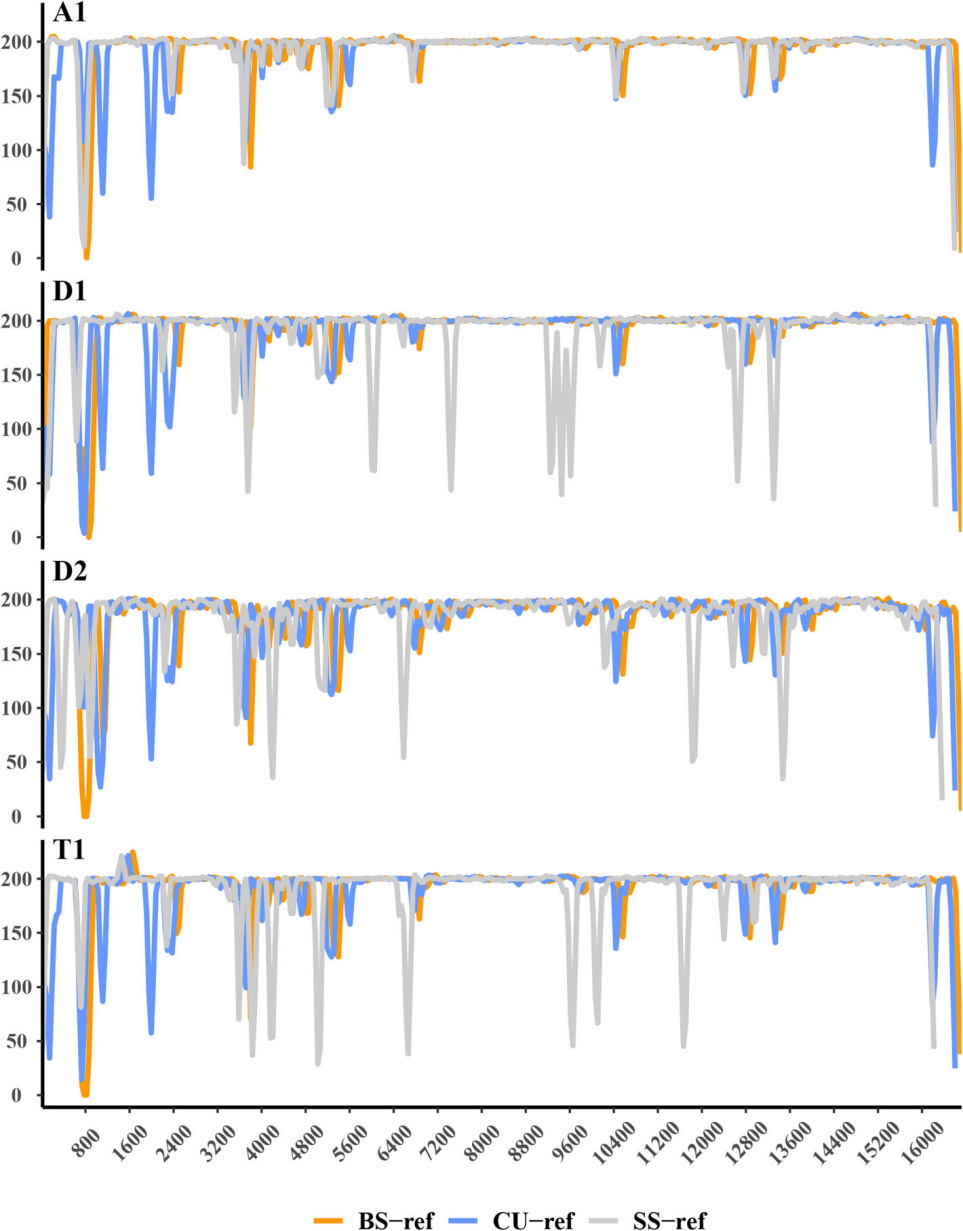
Comparisons of the mitogenome coverages mapping against the three reference sequences for each sample

Comparison the mitogenome quality obtained by mapping to different reference sequences showed that alignment against BS-ref yielded a higher mapping ratio and a larger average coverage than CU-ref or SS-ref, while the latter two performed similarly in mapping ratio and average coverage. In terms of time consumption during mitogenome alignment, mapping NGS data to BS-ref or to CU-ref nearly completed at the same time, but faster than to the SS-ref (Fig. 1). Detailed statistics of mapping ratio, coverage and CPU time for mitogenome mapping against the three different types of reference sequences are listed in Additional file 2: Table S2. Moreover, mapping against BS-ref showed a more uniform coverage across the mitogenome than against CU-ref or SS-ref (Fig. 2).

### Performance of SNP calling strategies on mitogenome diversity

As the gold standard, Sanger sequencing data for each specimen were aligned against reference sequence KP765605.1 for sample A1, KM044240.1 for D1 and D2, and KM073256.1 for T1. The number of SNPs was 12 for A1, 7 for D1, 9 for D2, and 10 for T1, which are detailed in Additional file 3: Table S3. A total of 18 SNP calling strategies, performed with three variant callers, i.e.*,* SAMtools 1.3.1 [24], VarScan 2.3.9 [25] and GATK 3.7 [26] with different parameter combinations (detailed in Table 2), were carried out to call SNPs using the bam files resulted from mitogenome alignments against the BS-ref sequences. All the SNPs we found were homogeneous substitutions compared to BS-ref. Analysis of the concordance between the SNP calling results from NGS and Sanger data revealed that all the eighteen strategies detected all true SNPs, with zero false positives and zero false negatives (see Additional file 4: Table S4).

**Table 2:** Summary of the different software packages used to call SNPs

Name	Software	Command	Option
**BCF**	SAMtools; BCFtools	mpileup; call	Default; −c
**BCF_B**	SAMtools; BCFtools	mpileup; call	-B; −c
**BCF_E**	SAMtools; BCFtools	mpileup; call	-E; −c
**HAP**	GATK	HaplotypeCaller	Default
**UNI**	GATK	UnifiedGenotyper	Default
**UNI1**	GATK	UnifiedGenotyper	-ploidy 1
**VAR_B01**	SAMtools; VarScan	mpileup; mpileup2snp	-B; −-min-var-freq 0.01
**VAR_B10**	SAMtools; VarScan	mpileup; mpileup2snp	-B; −-min-var-freq 0.10
**VAR_B25**	SAMtools; VarScan	mpileup; mpileup2snp	-B; −-min-var-freq 0.25
**VAR_B50**	SAMtools; VarScan	mpileup; mpileup2snp	-B; −-min-var-freq 0.50
**VAR_E01**	SAMtools; VarScan	mpileup; mpileup2snp	-E; −-min-var-freq 0.01
**VAR_E10**	SAMtools; VarScan	mpileup; mpileup2snp	-E; −-min-var-freq 0.10
**VAR_E25**	SAMtools; VarScan	mpileup; mpileup2snp	-E; −-min-var-freq 0.25
**VAR_E50**	SAMtools; VarScan	mpileup; mpileup2snp	-E; −-min-var-freq 0.50
**VAR01**	SAMtools; VarScan	mpileup; mpileup2snp	Default; −-min-var-freq 0.01
**VAR10**	SAMtools; VarScan	mpileup; mpileup2snp	Default; −-min-var-freq 0.10
**VAR25**	SAMtools; VarScan	mpileup; mpileup2snp	Default; −-min-var-freq 0.25
**VAR50**	SAMtools; VarScan	mpileup; mpileup2snp	Default; −-min-var-freq 0.50

## Discussion

In order to generate accurate genome sequences from NGS data, many studies have explored optimal alignment strategies [13–15, 27–29]. Both de novo and reference-based approaches were used in mitogenome reconstructions of *Clarias batrachus* from NGS data, resulting in consensus sequences with different lengths [13]. Moreover, different reference sequences led to different mapping performances. Liu et al. found that when the sample-specific sequence, i.e. a sequence with the same genotype sequence as the sample, was used as mapping reference in NGS analyses of HBV (Hepatitis B Virus), mapping accuracy and variant calling were optimized compared to the other four HBV sequences from the GenBank database commonly used as reference [14]. In general, a reference sequence belonging to the same species as the sample population, i.e., a reference sequence referred to as CU-ref sequence in this study, is the choice for sequence alignment [30–32]. However, reference genomes often cannot represent the full range of genetic diversity as a result of geographical divergence and independent demographic events of different populations. To comprehensively characterize genetic variation, different references for different populations are necessary. This perspective has motivated the research on pan-genomes, currently focusing on nuclear genomes, and not involving mitogenomes [16, 17].

In this study, we compared the mitogenome alignment quality obtained by mapping to three types of reference sequences, and proposed the optimal reference sequence for mtDNA analyses of specific populations. As Figs. 1 and 2 shown, alignment against BS-ref performed better in mapping ratio, average coverage and mitogenome coverage uniformity than against the other two kinds of reference sequences, and in the NGS data mapping process both BS-ref and CU-ref using have an advantage over SS-ref regarding computing time. Thus, the comparison above shows that the mapping strategy based on BS-ref showed a slightly better performance than the other two. This result was consistent with previous studies [27, 33]. Lee et al. revealed that the references with a closer genetic relationship to investigate *Mycobacterium tuberculosis* samples showed the highest proportion of reads that successfully aligned, which influenced the detection of mitochondrial SNP in later step [33]. To deeply analyze the basic reason in different mapping efficiency, we aligned the three kinds of reference sequences (CU-ref, BS-ref and SS-ref sequences) by MEGA7 [34], and the results were detailed in Additional file 5: Table S5. More than 200 polymorphic loci existed between CU-refs and the other two reference sequences. Polymorphisms between BS-ref and SS-ref were low-level, ranging from 9 to 38. The polymorphic difference among the reference sequences showed just right the difference of genetic relationships between references and samples, consistent with the statements of the three references. The reference sequence difference was a basis of difference in mapping efficiency, which devoted the importance of reference sequences for specific population during mitogenome alignment.

Mitogenome SNP detection is important, for example for functional annotation. A total of 18 different SNP calling strategies using three software programs with different options were compared, and led to the same SNP results in terms of true SNPs, false positives and false negatives. The results showed that the mitogenome obtained from the breed-specific alignment had a low requirement for variation calling tools and parameter choices, which indicated that mapping to the breed-specific reference sequence contributed to an accurate mitogenome. Therefore, breed-specific reference sequences were useful for mitogenome alignment of high reliability, and also worked well for the later detection of mitogenome variation. Though the mitogenome mapping against the BS-ref showed a slightly better performance in the mapping process, its performance in the SNP calling process was a plus, especially useful for NGS data of low depth [35], for example for the study of ancient DNA, by reducing erroneous base incorporations.

In addition, the de novo assembly comparison showed that the homologous read sets filtered by BLAST from clean data were suitable. The BLAST algorithm [36], proposed by Altshul et al. in 1990, is now the most widely used search tool for homologous sequences in nucleotide databases. Its tolerance for mismatches and gaps is better than that of BWA, which only identifies extremely stringent sequence similarities [37]. Inadequately, in this study, the de novo assemblies of the specimens were not complete containing some gaps, which might result in a slightly poor performance in the alignments to SS-ref sequences.

## Conclusions

In this study, different kinds of reference sequences, representing different genetic relationships to the investigated samples, were used in mitogenome analyses based on NGS data. The breed-specific sequence gave an optimal performance due to its high accuracy both in mitogenome mapping and SNP calling. Overall, this study underscored the importance of reference sequence choice in mitogenome research.

## Methods

### Animal ethics statement

All experimental pigs were maintained according to the guidelines of the experimental animal management of China Agricultural University. Animal management and experimental protocols complied with the guidelines approved by the Institutional Animal Care and Use Ethics Committee (IACUC) at China Agricultural University. After the study, the pigs were still living in the original environment.

### DNA sequencing

Ear tissues from four pigs of three breeds were collected, including an Asian wild boar (A1), two unrelated Diannan small-ear pigs (D1 and D2) and a Tibetan pig (T1). The pigs were all female except D1, and the ear samples were collected in their early adult life. Total DNA was extracted using the QIAamp DNA Investigator kit (QIAGEN, Hilden, Germany) following the manufacturer’s instructions. DNA quality was evaluated by spectrophotometry and agarose gel electrophoresis. DNA templates were ultrasonically sheared using a Covaris E220 (Covaris, Woburn, USA), and were converted into DNA libraries following the NEBNext Ultra DNA Library preparation protocol. Multiple Ampure Bead XP cleanups (Beckman Coulter, Brea, CA, USA) were conducted to remove any adapter dimers that might have developed. Quality and concentration of libraries were determined on an Agilent Bioanalyzer 2100 (Agilent Technologies, Santa Clara, CA). Subsequently, the quality-controlled genomic library for each sample was PE100 sequenced using the Illumina HiSeq 2000 sequencing system.

The traditional sequencing approach, Sanger sequencing, was also performed on the samples to represent the gold standard in variant detection. The mitogenome was PCR-amplified with 16 primer pairs used in a previous study [21]. Amplicons were bi-directionally sequenced using the BigDye Terminator version 3.1 technology on an ABI 3730 system (Applied Biosystems, Foster City, CA). Mitogenomes were analysed with the software packages MEGA6 [38] and DnaSP v5 [39].

### Quality control

Read quality was assessed using FastQC focusing on base quality scores and sequence length. Adapters and low-quality bases were removed with Clip&Merge. Reads shorter than 35 bp and a Phred quality score lower than 20 were removed. Next, forward and reverse reads were merged into single sequences if they overlapped by at least 8 bp. The above tools were used in an integrated pipeline, EAGER [40]. The filtered NGS data after the above steps were then used for downstream analyses.

### Alignment to different reference sequences

Mitogenome mapping was performed with BWA [41] with default parameters for the commands “aln” and “samse”. Three types of reference sequences regarding the genetic relationship between the reference sequences and the sample were used as follows.

1. The commonly used reference sequence (CU-ref), which referred to a frequently-used sequence from the RefSeq project in the NCBI database. Here NC_000845.1 was used, which is the mitogenome sequence from a Landrace [18–21].
2. The breed-specific reference sequence (BS-ref), which referred to a sequence of the same breed as the sample, and was downloaded from the NCBI database. For the Asian wild boar, KP765605.1 was used as BS-ref, which is from a Changbai mountains wild boar and 16,720 bp in length; for the Diannan small-ear pigs, KM044240.1 was used, which is a complete mitogenome of 16,720 bp obtained from a Diannan small-ear pig in Yunnan Province; and for the Tibetan pig, KM073256.1 was used, which is a Tibetan complete mitogenome of 16,710 bp.
3. The sample-specific reference sequence (SS-ref), which referred to the consensus sequence obtained from the NGS reads of the sample through de novo assembly.

The BAM files obtained from BWA were filtered for sequences with a mapping quality of at least 30. Duplicate reads that showed identical start and end coordinates were removed using DeDup. These tools were also integrated into the EAGER-pipeline [40].

The quality of mitogenome mapping was assessed by the mapping ratio, average coverage and run time. The mapping ratio refers to the ratio of reads mappable to the mitochondrial reference to all clean reads. Average coverage refers to the number of times the mitogenome is sequenced. Runtime refers to CPU time consumption during the mapping processing, instead of elapsed time, including waiting for input/output operations or entering low-power mode.

### De novo assembly for SS-ref construction

To produce the optimal SS-ref, three modified de novo assembly strategies were compared based on the NGS data from A1. They were different in the NGS read sets, including all clean read sets, homologous read sets filtered by BLAST, or BWA mapping. De novo assembly was performed using SOAPdenovo2 [22] with default parameters with the best k-mer size estimated by KmerGennie [23]. The detailed assembly information was as follows.

1. “Denovo”: the de novo assembly directly from all clean reads [23, 35]. All clean data from NGS were put into SOAPdenovo2 and contigs assembled. Then these contigs were aligned to NC_000845.1 using MEGA6, and a consensus inferred [35].
2. “BLAST_denovo”: the de novo assembly by homologous read sets filtered from clean data by BLAST. Clean data were filtered against a reference panel composed of all complete *Sus Scrofa* mitogenome sequences (219) downloaded from the NCBI database by the BLAST tool with the blastn command, and then these sets were put into SOAPdenovo2 for de novo assembly.
3. “BWA_denovo”: the de novo assembly by homologous read sets filtered from clean data by BWA. Clean reads were mapped against the above-mentioned reference panel to filter homologous sequences of each sample by BWA, and then these sequences were assembled by SOAPdenovo2*.*

To assess the three de novo assembly strategies, the indicators including the N50, consensus length, coverage and sequence polymorphism resulted from each strategy were measured.

### SNP calling of mitochondrial genomes

Three variation callers, i.e.*,* SAMtools 1.3.1 [24], VarScan 2.3.9 [25] and GATK 3.7 [26], were applied with different parameter combinations detailed in Table 2 to the bam files resulting from the mitogenome alignments. These parameters were selected to ensure comparability among different callers. The minimum base quality required to consider a base for calling was set to 30.

The performance of SNP calling was evaluated using the overall genotype concordance by comparing the NGS results with the Sanger data, with the assumption that the Sanger sequencing gave the correct calling [42–44]. Only positions where a Sanger sequence was available were kept, and SNPs concordant between Sanger and NGS data for each individual were considered as true SNPs, while discrepancies were considered as errors. When NGS data identified an alternate homozygote not observed by Sanger sequence, it was considered as a false positive. Accordingly, when NGS data did not see an alternate homozygote found with Sanger sequence, it was considered as a false negative. The number of true SNPs, false positives and false negatives were analysed.

## Supplementary Information

**Additional file 1: Table S1.** Sample information on whole-genome sequencing data.

**Additional file 2: Table S2.** Statistics of mapping ratio, coverage and CPU time cost for mitogenome mapping of NGS data.

**Additional file 3: Table S3.** SNP information resulting from Sanger sequences aligning to the breed-specific references.

**Additional file 4: Table S4.** The results of SNP calling for NGS data.

**Additional file 5: Table S5.** Polymorphic loci among the three kinds of reference sequences.

## Data Availability

The datasets analysed during the current study are available in the NCBI repository under the accession number PRJNA378496 including SRA544899 (A1), SRA544150 (D1), SRA544170 (D2) and SRA544142 (T1).

## Abbreviations

NGS: Next-generation sequencing
Mitogenome: Mitochondrial genome
MtDNA: Mitochondrial DNA
SNP: Single nucleotide polymorphism
NUMTS: Nuclear mitochondrial sequences
HBV: Hepatitis B virus
